# Objective Facts or Misleading Hype? Associations between Features of E-Cigarette Marketing and Sales on a Chinese E-Commerce Platform

**DOI:** 10.3390/ijerph17186711

**Published:** 2020-09-15

**Authors:** Yibei Chen, Shaojing Sun, Xiaoquan Zhao, Han Zhou, Fan Wang

**Affiliations:** 1 Department of Communication, University of California, Santa Barbara, CA 93106, USA; yibeichan@gmail.com; 2 School of Journalism, Fudan University, Shanghai 200433, China; shaojingsun@gmail.com (S.S.); zh.zhouhan.z.h@gmail.com (H.Z.); 3 Department of Communication, George Mason University, Fairfax, VA 22030, USA; xzhao3@gmu.edu; 4 Department of Politics, East China Normal University, Shanghai 200241, China

**Keywords:** electronic cigarettes, online marketing, content analysis

## Abstract

*Background*: Electronic cigarettes (e-cigarettes) have been increasingly advertised and marketed in China in recent years. This study examined the practice and impact of e-cigarette online marketing on a major retail website—Tmall.com. *Methods*: Data were obtained by crawling 449 online pages of e-cigarette marketing. Content analysis was conducted to summarize the marketing practices for four types of e-cigarettes, and multilevel modeling (MLM) was implemented to explore factors predictive of the online sales of the products. *Results*: The sales volume of e-cigarettes ranged from 0 to 28,169, with the price per item varying from RMB 218.1 ($31.84) to RMB 385.5 ($56.29). Fruit (44.3%, *n* = 199), mint (33%, *n* = 148) and cream/sugar/ice (29.4%, *n* = 132) were the three flavors most often listed for sale online. Moreover, 63.4% (*n* = 285) of e-cigarette ads emphasized the role of the products as an aid to quit smoking. Nice taste (75.1%), big vapor (65.7%), high capacity batteries (67.9%), fashionable models (61.3%), discounted price (49.7%), and suitability for gifting (45.9%) were the most frequently touted product features in online ads. Type of e-cigarettes, diversity of products, number of online comments, and location of manufacturers were significantly associated with sales volume. *Conclusions*: Online marketing of e-cigarettes was common on one of China’s leading e-commerce websites. Sellers employed advertising strategies targeting a wide range of potential consumers—from youth to the elderly. Stricter regulations of online marketing for e-cigarettes should be enforced in China.

## 1. Introduction

Formally termed electronic nicotine delivery systems (ENDS) and electronic non-nicotine delivery systems (ENNDS), electronic cigarettes (e-cigarettes) are battery-powered devices heating and aerosolizing a liquid solution for users to inhale [1,2]. Although e-cigarettes are often touted as a device to assist quitting conventional smoking, evidence on the effectiveness of using e-cigarettes for smoking cessation remains inconclusive [3]. Furthermore, there is increasing concern over the health risks associated with e-cigarette use [4,5]. Recently, some countries (e.g., the U.S.) have stepped up regulations over e-cigarettes out of concern for public health [6,7].

At the same time, e-cigarette producers have ramped up advertising and marketing of their products. As De Andrade and colleagues observed, marketing of e-cigarettes in the UK during 2012 and 2013 has been directed toward both the general public and policymakers. While misleading or overstated claims (e.g., “e-cigarettes are safe, healthy, and harmless.”) are used to attract consumers, statements about the products’ benefits for public health are weaved into marketing to influence policymakers. For example, an e-cigarette Facebook page stated that NICE (National Institute for Health and Care Excellence) had become the first public institution to recommend e-cigarettes for smokers who are unable to quit [8]. Researchers found that a large amount of online information featuring e-cigarette brands emphasizes the advantages (e.g., multiple flavors) of the products over conventional cigarettes [9,10]. Of more concern, however, is the prevalence of unsubstantiated claims (e.g., “E-cigarettes are a healthier alternative to conventional smoking.”) the industry have been employing to glamorize, advertise, and market e-cigarettes [11]. 

There has been a mounting call for regulating blatant marketing misinformation about e-cigarettes. According to Xinhua News Agency, the offcial state-run press agency of China, the main marketing avenue for e-cigarettes in China is the Internet, including e-commerce websites, social networking platforms, video-sharing sites, search engines, online forums, among others [12]. One study conducted in the U.S. in 2015, for example, examined about sixty e-cigarette retail websites and found a high prevalence of unfounded health claims or marketing claims, appealing to the youth but running counter to tobacco-control policies—stipulating that e-cigarettes be regulated as tobacco products, and misleading claims on the sites be prohibited [13]. Furthermore, studies found that exposure to e-cigarette marketing—Internet, print, retail, and TV/movies—significantly increased adolescents’ e-cigarette use [14,15,16]. For nonusers of e-cigarettes, increasing exposure to marketing messages could boost their likelihood of using the products in the future. Surveying 307 multiethnic college-students, Pokhrel, Fagan, Kehl, and Herzog reported that one’s receptivity to e-cigarette marketing was associated with a heightened perception of e-cigarettes as low-harm products, resulting in the more frequent use of e-cigarettes [17]. 

Housing the world’s largest population of conventional smokers, China has seen a rapid growth in the production and consumption of e-cigarettes over the past years. Chinese e-cigarette manufacturers have employed various marketing strategies to promote their products. While the industry is booming in China, regulation of e-cigarette marketing has been lax [18]. Yao and colleagues’ recent research found that e-cigarette manufacturers in China frequently overstate the benefits of the products—such as no secondhand smoking exposure and help for smoking cessation—while ignoring or downplaying potential health risks [19]. 

Different than Yao et al.’s study that focused on the manufacturers’ websites, the present study looks into a major retail website (hereinafter referred to as Tmall), the largest Business to Customer (B2C) platform in China. We chose Tmall for two main reasons: first, different from Alibaba.com (Business to Business) and Taobao.com (Customer to Customer), Tmall sells goods directly from the manufacturers (Business to Consumer; B2C) and provides more options for companies to customize their ads [20]; second, according to the China Internet Network Information Center, by March of 2020, the number of Internet users in China reached 904 million, with more than 78.6% of them having experience in online shopping [21]. Tmall is a primary venue for online shopping activities, accounting for 61.5% of China’s B2C E-commerce market [22].

The persuasive appeals of advertising have a significant influence on audiences’ attitudinal and behavioral responses to e-cigarette marketing [23]. For example, research showed that an emphasis on e-cigarettes’ lower cost, greater healthfulness, and utility compared to regular cigarettes could boost smokers’ intention to try e-cigarettes [24]. Moreover, ads with the presence of models using e-cigarettes elicited more interest than those without [24]. Researchers found that even passive exposure to e-cigarette visual imagery can increase smoking desire and e-cigarette use in young adults [25,26,27,28,29]. The present study explores the characteristics of e-cigarette marketing on Tmall, the largest online retail platform in China, and investigates the relationship between advertising features and the actual sales of the products. As such, this study seeks to shed light on the marketing strategies employed by e-cigarette manufacturers and retailers, as well as the potential effects of these strategies on product purchases. The following research questions guide our exploration:

Research Question 1: What are the characteristics of the e-cigarettes ads on the retail website?

Research Question 2: What are the impacts, if any, of ad features on the actual online sales?

## 2. Materials and Methods

### 2.1. Data Collection

We conducted a search on Tmall on December 30th, 2018, using the keyword “e-cigarettes” (in Chinese), which yielded 4400 results. After dropping 2684 accessories (e.g., atomizing cores, aerosols, chargers, batteries, cables, e-liquids, stickers, etc.), 1716 products were retained. Because of the highly skewed distribution of sales volume, we stratified the data into two strata by using the inflection point of the distribution as a threshold. Specifically, one stratum included all products with sales volume higher than or equal to 24 (*n* = 306), and the other stratum included 306 randomly sampled products from the 1410 with sales volume lower than 24. Since products and product information are regularly updated on Tmall, we repeated the search on 1 February 2019 to verify product status and filter out short-lived products. Products that did not appear in the second search (*n* = 163) were excluded from a nalysis. A total of 449 e-cigarette products were retained in our final sample.

Metadata for each product were crawled and stored through a python script (with the package selenium 3.141, which is a reliable, open-source tool that can send standard Python commands to different browsers, despite variation in browser design [30]), including product names, links, prices, sales volumes, comments from customers who made online purchases, etc. Moreover, considering each seller on Tmall can sell multiple e-cigarette products, we scraped the metadata of each seller (name, link, location, reputation, etc.) to capture the nested structure between sellers and products. 

### 2.2. Coding Procedure

Previous studies [15,31,32] were consulted to develop a codebook to capture the following textual and visual elements in ads: (1) brand type (overseas vs. domestic); (2) product type (cigalike, which mimics all the design features of real cigarettes, down to the filter pattern and the ash-like LED tips that lights up red when activated; vape pen, which has a pen-like shape and provides more flexible vaping experience than a cigalike; vape pod, which combines the portability and ease of use of a vape pen or cigalike with the power of a mod; or vape mod, which is the largest and the most powerful among the four types, and produces more vapor); (3) shopping options (selections of size, color, set, etc., provided with the product); (4) first-screen content (the main information presented on the first screen); (5) health as being environmentally friendly and no tar/CO/carcinogen/secondhand smoke; (6) cessation claims such as cigarette replacement/cessation/reduction; (7) user-experience claims such as good taste/smell/feel, enjoyable, big vapor, portable, easy to use; (8) product-quality claims such as safety protection and dustproof; (9) venue of use such as indoor, outdoor, travel; (10) Money saving or economical; (11) type of flavors such as soft drink, alcohol, sweets/candy, menthol, fruit; (12) promotions such as gift promotions, warranty; (13) individual benefits such as symbol of social status, freedom, individualism, and wisdom; (14) social benefits such as improving romantic/family/interpersonal relationship; (15) gift giving such as “the e-cigarette is a perfect gift for father/friend/colleague”; (16) warnings such as “e-cigarettes are not safe for youth, young adults, pregnant women, as well as adults who do not currently use tobacco products”; (17) models or celebrities in ads such as gender, number of models. Except for the brand type for which 0 represents domestic and 1 represents overseas, all other items were coded as 0 (“absent”) and 1 (“present”), and scores were aggregated for each category. For example, health claims would be scored 5 if there were five non-repeated health-related claims in one ad. 

With a random sub-sample of 61 (10% of the total *n*) items, two coders conducted a pilot content analysis, with Krippendorff’s alpha ranging from 0.71 to 0.83 for all the coded variables. After the discussion and resolution of discrepancies, the coders reached agreement on the coding schemes and proceeded to divide the remaining 388 items and finished coding independently.

### 2.3. Data Analysis

We first conducted descriptive analyses and Chi-Square tests for coded data to explore whether ad features vary across product types. Since products were nested within sellers, we applied random intercept multilevel models (MLM) to explore potential predictors of online sales of e-cigarettes, with ads features and product metadata as level-1 predictors and sellers’ information as level-2 predictors. The outcome variable was sales volume denoting the number of products sold during the past 30 days. Due to its highly skewed distribution (see Table 1), a log transformation was applied to sales volume. The level-1 predictor variables included brand type, product type, shopping options, first-screen content, claims (health-related, cessation-related, quality-related, user experience-related), venue of use, gift-giving, individual benefits, social benefits, promotions, number of models/celebrities, price (log transformed), and number of comments (log transformed). On level-2, we added another two predictors: location of the shop, service score of the shop (none, negative, or positive). For the log transformations, we added 1 to the original data points to be able to include data points having the undefined value of log(0) (*n* = 25 for sales volume and 16 for comments). All data cleaning, descriptive statistics, and Chi-Square tests were implemented with Python 3.7 (Python Software, DE, United States) (packages pandas 1.0.0 and scipy 1.2.1) and multilevel modeling was conducted with R 3.5.2 (R foundation, Vienna, Austria) (package lme4 1.1-21).

## 3. Results

Table 1 presents descriptive information about the sample, including sales volume, product price, and the number of customer comments for each product from the high (sales volume higher than 24) and low (sales volume lower than 24) strata, respectively. On the whole, products in the high strata tended to have a lower price and more comments. Sales volume across the four e-cigarette types (i.e., vape mod, vape pen, vape pod, and cigalike) ranged from 0 to 28,169 (*Mean* = 497.78, *SD* = 1909.01). The average price of the sampled products was 294.17 RMB ($42.9), with vape mod costing about 385.5 RMB ($54.4) per item, vape pen 218.1 RMB ($30.8), vape pod 235.3 RMB ($33.2), and cigalike 258.3 RMB ($36.5). Vape pens received an average of 3329 comments—followed by cigalikes (2734.57) and vape mods (2720.91), while vape pods received the least comments (967).

### 3.1. Content Analysis

Presented in Table 2 are content features of online ads by e-cigarette type. Of the 449 e-cigarette products from 165 online sellers, there were 169 vape mods, 137 vape pods, 87 vape pens, and 56 cigalikes. As for flavor, fruit (44.3%, *n* = 199) accounted for the largest share, followed by menthol (33%, *n* = 148) and sweets/candy (29.4%, *n* = 132). 

Claims of smoking cessation were common in e-cigarettes ads, with 63.4% (*n* = 285) of the ads emphasizing the products as being helpful for quitting smoking, 5.6% (*n* = 25) mentioning the rapidity of quitting smoking with the products, and 3.1% (*n* = 14) stating the products would cause no addiction. Some examples of health-related claims were “ingredients are plant fiber” (19.4%) or “eco-friendly” (14.7%), “e-cigars are tar/CO/carcinogen-free” (37.2%), “smoking e-cigars is healthy” (45.9%), and “no secondhand smoking” (28.7%). Chi-square tests showed that the ads of vape mods presented fewer health-related claims, compared with those ads of vape pods, pens and cigalikes.

Regarding user experience, a high percentage of ads (75.1%) stressed taste, while fewer ads (3.1%) mentioned the smell. In addition, ads about vape mods mostly emphasized big vapor (65.7%) and the fun of playing with vapor (28.4%). There was a statistically significant difference in the frequency of claims about the quality of e-cigarette products (χ^2^(3) = 156.79, *p* < 0.001). Across all products, claims about the high capacity of batteries appeared most frequently (67.9%), followed by claims about safety protection (59.0%), leakproof (52.6%), and high-tech (50.1%). Compared with the other three types, ads of vape mod put more emphasis on product quality, especially high power (77.5%).

Despite a lack of statistically significant difference, models/celebrities appeared more often in ads (61.3%) about vape mods. Of all 299 ads with models, 132 used both male and female models, 50 with only females, and 114 with only males. Furthermore, models in most ads (96.0%) were young adults ages 18–35.

With regard to social benefits, 16.5% of the ads mentioned family harmony and 8.2% mentioned interpersonal relationships such as building relationships with colleagues, increasing friendship, etc. Also, 5.6% of the ads mentioned romantic relationships. As for individual benefits, 20% of the ads conveyed the message that consuming e-cigarettes is a symbol of social status, 17.4% connected e-cigarettes to unrestrained joy and freedom, and 16.5% claimed that the products represent individualism. Furthermore, 16.9% of the ads said that e-cigarettes could be a good choice for gift-giving.

Almost half of the ads (49.7%) mentioned discounts for purchasing whole packages and 45.9% offered stickers or e-liquids as free gifts. Sixty percent (60%) of the products carried a warranty. Only 37.9% of the ads warned that the products were not suitable for juveniles, pregnant women, patients with certain diseases (e.g., severe cardiovascular diseases, diabetes) or nicotine allergy.

### 3.2. MLM Results

For the null MLM model, results revealed an intraclass correlation coefficient (ICC) of 0.23, evidencing the suitability of a nested-design analysis. Moreover, we applied residual normality test to the conditional models, with Q-Q plots and the Shapiro Test (*p_level-1 model_* = 0.35 > 0.05, *p_level-2 model_* = 0.66 > 0.05) suggesting the tenability of normality. In other words, our analysis did not violate the normality assumption.

As shown in Table 3, in the level-1 model, among the four types of e-cigarettes, the Vape pod was the most popular (*b* = 0.57, *p* < 0.01). Also, products with more selections (*b* = 0.06, *p* < 0.001) landed more sales. Compared to other ads claims, health-related claims had a stronger relationship with product sales. Each additional health claim was associated with an 18.5% increase in sales volume. The order of the claims presented on the page did not significantly correlate with sales. As expected, the number of online comments had a strong positive relationship (*b* = 0.57, *p* < 0.001) with sales volumes—that is, a 10% increase in the number of comments was associated with 5.6% greater sales volume. Price was negatively related to sales (*b* = −0.41, *p* < 0.001), with each 10% increase in price relating to a 4.58% decrease in sales. The level-2 model showed that overseas sellers (*b* = 3.03, *p* < 0.01) had 1969.7% greater sales volumes than domestic sellers. However, other seller features such as service scores had no significant associations with sales.

## 4. Discussion

To our best knowledge, the present study—analyzing ads of 449 products within 165 shops—is the first to focus on the marketing of e-cigarettes on the largest Chinese e-commerce platform, Tmall. With bans on indoor smoking becoming tighter across China, marketers try to portray e-cigarettes as healthy products to facilitate quitting smoking. Smoking-cessation claims are common in the ads, followed by claims emphasizing e-cigarettes’ safety. While advertising of flavored e-cigarettes is widespread, other features (e.g., health-claims, user experience, and the use of models/celebrities) are emphasized differently across types of products. Furthermore, among all these features, health claims, product-quality claims, use of models/celebrities, and sellers’ location (domestic or overseas) are significantly associated with sales volume. Unsurprisingly, the number of comments from other customers and the price significantly contribute to sales volume as well.

It is still in question whether e-cigarettes safer alternatives to conventional cigarettes and effective gateways toward smoking cessation [33]. One point of contention is that the nicotine salts found in e-cigarettes are often described as non-nicotine ingredients, which is not the case [2]. In our study, 20.7% ads mentioned nicotine salts with vague claims about the product’s low nicotine content. Research found that the “5% nicotine” solution mentioned in JUUL ads actually reached a nicotine level between 56.2 and 69 mg/mL [34]. Researchers also tested 35 e-liquid samples labeled as 18 mg/mL nicotine and found 35–52% greater nicotine content than advertised [35].

Our study found that nearly every product is accompanied by more than five kinds of e-liquids, primarily fruit, sweets/candy, and soft drink flavors. The National Youth Tobacco Survey (NYTS) showed that 31% of American youth considered menthol, candy, fruit, or chocolate flavors of e-cigarettes to be the main reasons for use [36]. In August 2018, the State Administration for Market Regulation (SAMR) and the State Tobacco Monopoly Administration (STMA, also known as China Tobacco) jointly issued a ban on selling e-cigarettes to minors [37]. However, only 37.9% of the products’ ads mentioned prohibited youth access, and nearly all of the related warnings were placed in inconspicuous positions in the ads.

Our study compared different advertising strategies for four types of e-cigarettes. Products with models/celebrities in the ads sold better than those without. Pictures of glamorous models are more likely to appeal to youngsters, who actively chase fashion and popularity. The models in the ads for vape pens mostly featured a rebellious and rock style, wearing peaked caps, extensive tattoos, and exaggerated earrings. Ads of cigalike products featured more health and smoking-cessation claims, especially no secondhand smoking for families. Often, an ad would show a male model vaping and enjoying time with family along with his children or grandchildren. Ads of vape pens emphasized user experience as enjoyable and fashionable. In contrast, ads of vape pods often highlighted favorable social norms or expectations (e.g., consuming e-cigarettes is a sign of success in life). These findings were consistent with prior studies of e-cigarettes brands, showing that older brands (cigalike) tended to stress health and economic advantages, while newer brands (mostly vape pens and mods) tended to emphasize diverse options such as new flavors appealing to the youth [6,38]. 

Our findings have theoretical implications for both tobacco control and advertising research. First, as one of the few studies that examine e-cigarette marketing strategies in the Chinese context, this study adds valuable perspectives on the unique challenges of tobacco control in China and highlights the importance of contextualizing theory building to guide future intervention efforts in global settings. Second, our results suggest that advertising features are indeed predictive of online purchasing behavior in e-cigarette marketing. Additional research guided by both persuasion and behavioral theories should look further into the influence mechanisms in the relationships observed in this study.

The present study also offers practical implications to policymakers. In October 2019, the Chinese government issued “Notice on Further Protecting Minors from E-cigarettes,” urging the banning of e-cigarette advertising and the selling of e-cigarettes on e-commerce platforms and websites [39]. However, online marketing of e-cigarettes in China continues to operate on different platforms, such as e-cigarette forums and social media (e.g., WeChat). The marketing of e-cigarettes has become more covert, subtle, and elusive. On this note, the present study provides a first glimpse into the impact of online ads on the actual sales of e-cigarettes. More studies are warranted to continue to monitor manufacturers’ online marketing practices so as to assist public health professionals in designing effective counter-marketing strategies. Furthermore, the large effect of health claims—such that each additional health claim was associated with 18.5% greater sales volume—might suggest that regulations are required for health claims on e-cigarette ads to avoid misleading consumers. For example, invalid health claims should be banned on e-cigarette ads. Finally, our finding that a 10% increase in price was associated with 4.58% lower sales volume echoes the study by the European Union—which found that a 10% increase in prices was associated with 8.2% fewer e-cigarettes sales [40]. In this regard, our study informs policymakers about the value of excising taxes to reduce the consumption of e-cigarettes.

Several caveats should be noted with the findings. First, our study did not cover all e-cigarette products presented on the website, but rather a stratified sample with randomly drawn items from the lower sales stratum. Future studies can use a big-data technique to analyze much larger volumes of e-cigarette ads. Second, we recorded sales volume with a one-month time interval, which is reasonable but not perfect. Future studies, if possible, should investigate a longer period of sales and look into the potential impact of outside-the-platform factors (e.g., policy, public health campaign) on sales. Third, this study only examined one particular retail website, and its findings may not generalize to other online platforms. Lastly, Yao et al.’s study found that claims—for example, e-cigarettes are a smoking cessation aid, suitable for smoking anywhere, clean, environmentally friendly, and safer than conventional cigarettes—appear more frequently on Chinese websites, while other types of claims—such as e-cigarettes are economical, symbolic of social status, and modern—appear more frequently on English websites [19]. In this regard, comparative research across cultures would be promising.

## 5. Conclusions

Overall, this study showed that online marketing of e-cigarettes was widespread in China’s e-commerce marketplace. E-cigarette producers employed various marketing strategies to attract consumers’ attention and interest. Our results show that certain features of e-cigarette ads were significantly associated with sales volume on Tmall. Notable predictors included the use of models/celebrities, health claims, and product-quality claims. Strict regulations of online marketing for e-cigarettes should be enforced in China to safeguard public health. As an exploratory study, the current research has important limitations and is rather modest in scale. Future studies should look to employ stronger designs and more powerful data tools to further extend the current findings.

## Figures and Tables

**Table 1:** Sales volume, price, and number of comments for sampled products.

Descriptive Statistics	Sales Volume		Price		Number of Comments	
	High	Low	High	Low	High	Low
Mean	907.21	6.05	¥236 ($34.38)	¥371.84 ($54.16)	3924.83	361.20
Std	2514.10	6.21	¥171.32 ($24.95)	¥347.74 ($50.65)	10,214.73	1100.87
min	26	0	¥9 ($1.31)	¥ 35 ($5.10)	20	0
25%	58	1	¥128 ($18.64)	¥175.75 ($25.60)	211	6
50%	137	3	¥178 ($25.93)	¥288.5 ($42.02)	872	31
75%	579	11	¥298 ($43.41)	¥399.25 ($58.16)	3234	163
Max	28,169	23	¥1188 ($173.05)	¥2888 ($420.67)	96,471	11,451
Skewness	6.73	0.97	1.83	3.40	6.03	6.70

**Table 2:** Content features of online advertisements by e-cigarette type.

Features	Vape Mods (*n* = 169)		Vape Pods (*n* = 137)		Vape Pens (*n* = 87)		Cigalikes (*n* = 56)		Total (%)	χ^2^ *p*
	*n*	% (95% CI)	*n*	% (95% CI)	*n*	% (95% CI)	*n*	% (95% CI)		
**Cessation Claims**										
Cessation/control/replace	87	51.4 (47.6–55.3)	91	66.4 (62.4–70.5)	62	71.3 (66.4–76.1)	45	80.4 (75.0–85.7)	285(63.4)	0.088
Cessation progression	3	1.8 (0.8–2.8)	8	5.8 (3.8–7.8)	11	12.6 (9.1–16.2)	3	5.4 (2.3–8.4)	25(5.6)	0.023
No addiction	3	1.8 (0.8–2.8)	5	3.6 (2.0–5.2)	4	4.6 (2.4–6.8)	2	3.6 (1.1–6.1)	14(3.1)	0.753
**Health Claims**										
Healthy	53	31.4 (27.8–34.9)	72	52.6 (48.3–56.8)	47	54.0 (48.7–59.4)	34	60.7 (54.2–67.2)	206(45.9)	0.021
No tar/CO/carcinogen	32	18.9 (15.9–21.9)	71	51.8 (47.6–56.1)	36	41.4 (36.1–46.7)	28	50.0 (43.3–56.7)	167(37.2)	<0.001
Nicotine content	9	5.3 (3.6–7.1)	70	51.1 (46.8–55.4)	7	8.0 (5.1–11.0)	7	12.5 (8.1–16.9)	93(20.7)	<0.000
No secondhand smoking	30	17.8 (14.8–20.7)	44	32.1 (28.1–36.1)	30	34.5 (29.4–39.6)	25	44.6 (38.0–51.3)	129(28.7)	<0.01
Plant fiber	15	8.9 (6.7–11.1)	30	21.9 (18.4–25.4)	19	21.8 (17.4–26.3)	23	41.1 (34.5–47.6)	87(19.4)	<0.000
Eco-friendly	14	8.3 (6.2–10.4)	25	18.2 (14.9–21.5)	18	20.7 (16.3–25.0)	9	16.1 (11.2–21.0)	66(14.7)	0.142
**User Experience Claims**										
Taste good	131	77.5 (74.3–80.7)	111	81.0 (77.7–84.4)	60	70.0 (64.0–73.9)	35	62.5 (56.0–69.0)	337(75.1)	0.425
Smell good	2	1.2 (0.4–2.0)	7	5.1 (3.2–7.0)	3	3.4 (1.5–5.4)	2	3.6 (1.1–6.1)	14(3.1)	0.507
Feel good	80	47.3 (43.5–51.2)	27	19.7 (16.3–23.1)	20	23.0 (18.5–27.5)	22	39.3 (32.8–45.8)	149(33.2)	<0.005
Big vapor	111	65.7 (62.0–69.3)	21	15.3 (12.3–18.4)	51	58.6 (53.3–63.9)	13	23.2 (17.6–28.9)	196(43.7)	<0.000
Enjoyable	20	11.8 (9.3–14.3)	62	45.3 (41.0–49.5)	18	20.7 (16.3–25.0)	13	23.2 (17.6–28.9)	113(25.2)	<0.000
Play with vapor	48	28.4 (24.9–31.9)	7	5.1 (3.2–7.0)	14	16.1 (12.2–20.0)	2	3.6 (1.1–6.1)	71(15.8)	<0.000
Portable	89	52.7 (48.8–56.5)	102	74.5 (70.7–78.2)	48	55.2 (49.8–60.5)	45	80.4 (75.0–85.7)	284(63.3)	0.033
Easy to use	74	43.8 (40.0–47.6)	101	73.7 (70.0–77.5)	48	55.2 (49.8–60.5)	41	73.2 (67.3–79.1)	264(58.8)	0.016
**Quality Claims**										
Intelligent/high-tech	96	56.8 (53.0–60.1)	49	35.8 (31.7–39.9)	43	49.4 (44.1–54.8)	37	66.1 (59.7–72.4)	225(50.1)	0.024
High-power	131	77.5 (74.3–80.7)	35	25.5 (21.8–29.3)	31	35.6 (30.5–40.8)	6	10.7 (6.6–14.8)	203(45.2)	<0.000
Battery capacity	118	69.8 (66.3–73.3)	93	67.9 (63.9–71.9)	57	65.5 (60.4–70.6)	37	66.1 (59.7–72.4)	305(67.9)	0.98
Safety protection	120	71.0 (67.5–74.5)	64	46.7 (42.5–60.0)	46	52.9 (47.5–58.2)	35	62.5 (56.0–69.0)	265(59.0)	0.138
Dustproof	32	18.9 (15.9–21.9)	31	22.6 (19.1–26.2)	39	44.8 (9.5–50.2)	28	50.0 (43.3–56.7)	130(29.0)	<0.000
Leakproof	81	47.9 (44.1–51.8)	68	50.0 (45.4–53.9)	51	58.6 (53.3–63.9)	36	64.3 (57.9–70.7)	236(52.6)	0.367
Heatproof	62	36.7 (33.0–40.4)	9	6.6 (4.5–8.7)	23	26.4 (21.7–31.2)	8	14.3 (9.6–19.0)	102(22.7)	<0.000
**Venue of Use**										
In public	6	3.6 (2.1–5.0)	21	15.3 (12.3–18.4)	10	11.5 (8.1–14.9)	5	8.9 (5.1–12.7)	42(9.4)	0.061
Indoor	14	8.3 (6.2–10.4)	41	30.0 (26.0–33.8)	14	16.1 (12.2–20.0)	11	19.6 (14.3–25.0)	80(17.8)	<0.005
Outdoor	1	0.6 (0–1.2)	5	3.6 (2.0–5.3)	1	1.1 (0–2.3)	1	1.8 (0–3.6)	8(1.8)	0.406
For trip	7	4.1 (2.6–5.7)	32	23.4 (19.7–27.0)	9	10.3 (7.1–13.6)	5	8.9 (5.1–12.7)	53(11.8)	<0.001
Everywhere	13	7.7 (5.6–9.7)	36	26.3 (22.5–30.0)	10	11.5 (8.1–14.9)	11	19.6 (14.3–25.0)	70(15.6)	<0.01
Money-Saving	6	3.6 (2.1–5.0)	13	9.5 (7.0–12.0)	7	8.0 (5.1–11.0)	13	23.2 (17.6–28.9)	39(8.7)	<0.001
**Flavor**										
Soft drink	47	27.8 (24.3–31.3)	32	23.4 (19.7–27.0)	16	18.4 (14.2–22.5)	9	16.1 (11.2–21.0)	104(23.2)	0.280
Alcohol	7	4.1 (2.6–5.7)	11	8.0 (5.7–10.4)	5	5.7 (3.3–8.2)	8	14.3 (9.6–19.0)	31(6.9)	0.058
Sweets/candy	54	32.0 (28.4–35.5)	49	35.8 (31.7–39.9)	16	18.4 (14.2–22.5)	13	23.3 (18.7–27.7)	132(29.4)	0.074
Menthol	46	27.2 (23.8–30.6)	63	46.0 (41.7–50.2)	19	21.8 (17.4–26.3)	20	35.7 (29.3–42.1)	148(33)	0.017
Other spices	10	5.9 (4.1–7.7)	8	5.8 (3.8–7.8)	3	3.4 (1.5–5.4)	3	5.4 (2.3–8.4)	24(5.3)	0.849
Fruit	73	43.2 (39.4–47.0)	78	56.9 (52.7–61.2)	26	29.9 (25.0–34.8)	22	39.3 (32.8–45.8)	199(44.3)	0.031
Chinese tobacco	34	20.1 (17.0–23.2)	12	8.8 (6.3–11.2)	16	18.4 (14.2–22.5)	15	26.8 (20.9–32.7)	77(17.1)	0.030
Other tobacco	25	14.8 (12.1–17.5)	76	55.5 (51.2–59.7)	6	6.9 (4.2–9.6)	11	19.6 (14.3–25.0)	118(26.3)	<0.000
**Gift promotions**										
Stickers/liquids/etc.	82	48.5 (44.7–52.4)	53	38.7 (34.5–42.8)	42	48.3 (42.9–53.6)	29	51.8 (46.4–57.1)	206(45.9)	0.563
Package discount	88	52.1 (48.2–55.9)	60	43.8 (39.6–48.0)	43	49.4 (44.1–54.8)	32	57.1 (50.5–63.8)	223(49.7)	0.610
Warranty	103	60.9 (57.2–64.7)	72	52.6 (48.3–56.8)	52	59.8 (54.5–65.0)	41	73.3 (67.3–79.1)	268(59.7)	0.309
**Individual Benefits**										
Symbol of social status	26	15.4 (12.6–18.2)	32	23.4 (19.7–27.0)	14	16.1 (12.2–20.0)	18	32.1 (25.9–38.4)	90(20)	0.039
Freedom	39	23.1 (19.8–26.3)	14	10.2 (7.6–12.8)	18	20.7 (16.3–25.0)	7	12.5 (8.1–16.9)	78(17.4)	0.071
Individualism	34	20.1 (17.0–23.2)	17	12.4 (9.6–15.2)	19	21.8 (17.4–26.3)	4	7.1 (3.7–10.6)	74(16.5)	0.027
Wisdom	1	0.6 (0–1.2)	5	3.6 (2.0–5.3)	1	1.1 (0–2.3)	1	1.8 (0–3.6)	8(1.8)	0.406
**Social Benefits**										
Romantic relations	7	4.1 (2.6–5.7)	11	8.0 (5.7–10.4)	3	3.4 (1.5–5.4)	4	7.1 (3.7–10.6)	25(5.6)	0.445
Family harmony	10	5.9 (4.1–7.7)	28	20.4 (17.0–23.9)	16	18.4 (14.2–22.5)	19	33.9 (27.6–40.3)	73(16.3)	<0.001
Interpersonal relations	8	4.7 (3.1–6.4)	15	10.9 (8.3–13.6)	7	8.0 (5.1–11.0)	7	12.5 (8.1–16.9)	37(8.2)	0.271
**Gift Giving**	12	7.1 (5.1–9.1)	24	17.5 (14.3–20.8)	18	20.7 (16.3–25.0)	22	39.3 (34.0–44.5)	76(16.9)	<0.000
**Taboos**	65	38.5 (34.7–42.2)	61	44.5 (40.3–48.8)	21	24.1 (19.6–28.7)	23	41.1 (35.8–46.3)	170(37.9)	0.089
**Model/Celebrity**	76	45.0 (39.6–50.3)	84	61.3 (56.1–66.5)	49	56.3 (51.0–61.6)	42	41.1 (35.8–46.3)	251(55.9)	0.153

**Table 3:** Multilevel modeling predicting sales volume from the product, promotion, and seller characteristics.

Variables	Unstandardized Estimate (Standard Error)		
	Null Model	Level-1 Model	Level-2 Model
**Fixed Effects**			
Intercept	3.66 (0.14) ***	1.41 (0.78)	1.18 (0.85)
Oversea Brand		0.75 (0.17) ***	0.73 (0.17) ***
Shopping Option		0.06 (0.02) ***	0.06 (0.02) ***
Product Type			
Vape Mod (dummy)			
Vape Pod		0.57 (0.21) **	0.59 (0.21) **
Cigalike		0.51 (0.25) *	0.52 (0.26) *
Vape Pen		0.36 (0.21) *	0.34 (0.21)
First-Screen Content			
Design (dummy)			
Experience		−0.05 (0.31)	−0.13 (0.33)
Quality		−0.10 (0.28)	−0.16 (0.30)
Health		−0.01 (0.30)	−0.03 (0.34)
Promotions		−0.25 (0.26)	−0.27 (0.25)
Benefits		0.34 (0.38)	0.51 (0.38)
Cessation Claims		0.12 (0.14)	0.08 (14)
Health Claims		0.17 (0.06) **	0.17 (0.06) **
Quality Claims		0.13 (0.05) *	0.11 (0.05) *
User-experience Claims		−0.06 (0.06)	−0.05 (0.06)
Venue of Use		−0.15 (0.06) *	−0.14 (0.07) *
Gift Giving		−0.19 (0.22)	−0.22 (0.22)
Model/celebrity		0.50 (0.15) ***	0.52 (0.16) ***
Individual Benefits		0.19 (0.10) *	0.19 (0.10)
Social Benefits		−0.03 (0.13)	0.03 (0.06)
Promotions		0.03 (0.06)	0.03 (0.06)
Log (Price)		−0.41(0.11) ***	−0.40 (0.11) ***
Log (# of Comments)		0.57 (0.03) ***	0.79 (0.03) ***
Shop Location			
Domestic (dummy)			
Overseas			30.03 (10.47) *
Shop Service Score			
None (dummy)			
Negative			0.31 (0.31)
Positive			0.19 (0.27)
**Random Effects**			
Shop Intercept			0.29 (0.54)
**ICC**	0.23	0.14	0.14

Note. *** *p* < 0.001, ** *p* < 0.01, * *p* < 0.05
